# Lost in Thought or Just Lonely? Everyday Cognitive Competence in Middle Adulthood

**DOI:** 10.3390/ejihpe15040058

**Published:** 2025-04-10

**Authors:** Luka Juras, Marina Martincevic, Andrea Vranic

**Affiliations:** Department of Psychology, Faculty of Humanities and Social Sciences, University of Zagreb, 10000 Zagreb, Croatia; ljuras@ffzg.unizg.hr (L.J.); mmartincevic@ffzg.unizg.hr (M.M.)

**Keywords:** everyday cognitive competence, loneliness, executive functions, middle-aged adulthood

## Abstract

Everyday cognitive competence refers to the ability to manage cognitively demanding tasks essential for maintaining functional independence. While cognitive abilities are well explored in explaining individual differences in everyday cognitive competence, growing attention has been directed toward the impact of non-cognitive factors like loneliness. This study aims to investigate how executive function (EF) components—updating, inhibition, and task shifting—predict everyday cognitive competence and whether loneliness explains the additional variance beyond EF processes. To account for the multifaceted nature of everyday cognitive competence, both performance-based (Everyday Problems Test—EPT) and self-reported measures (Cognitive Failures Questionnaire—CFQ) were administrated. The sample included 176 middle-aged adults (ages 43–65), a group suitable for investigating predictors of everyday cognitive competence in the early stages of cognitive aging. The findings reveal that updating is a significant predictor of the performance on the EPT, while loneliness is not. When self-reported cognitive lapses are considered, loneliness emerges as a significant predictor. The lack of a relationship between the EPT and CFQ, along with their differing associations with EF, loneliness, and sociodemographic factors, suggests they assess distinct aspects of everyday cognitive competence. This highlights the need for a multidimensional assessment framework to gain a comprehensive understanding of everyday cognitive competence in middle-aged adults.

## 1. Introduction

The aging of population, combined with the increasing complexity of daily life, has made the safeguarding of functional independence in older adulthood a vital and pressing concern. This concern extends beyond the older population, influencing healthcare practices and societal planning. One concern is cognitive decline, a well-documented phenomenon associated with aging, particularly in fluid abilities, such as working memory (WM), executive function (EF), reasoning, and processing speed (Glahn et al., 2013). Evidence suggests that these changes may begin as early as midlife, with some EF processes starting to decline around 35 years of age (Ferguson et al., 2021). This decline typically follows a gradual and linear trajectory over time, with fluid abilities being more strongly correlated with age than most other individual characteristics in psychology. For example, processing speed and age have a correlation of -0.47 (Salthouse, 2004), while WM performance declines by approximately one standard point between the ages of 50 and 69 (Alloway & Alloway, 2013). Rather than focusing exclusively on older adults, recognizing the early decline onset is important for understanding the trajectory of cognitive changes that influence everyday functioning across the lifespan. Importantly, a decline in fundamental cognitive processes only partially explains age-related changes in everyday cognitive competence.

Everyday cognitive competence, defined as the ability to manage cognitively complex daily tasks essential for independent living (Willis, 1996), is central to cognitive aging research. Studies involving older adults often explore cognitive competence to identify deficits in these skills and uncover challenges in daily functioning, aiming to develop targeted support and interventions. However, its significance extends beyond aging populations, as these skills are critical for functioning effectively in daily life and contributing to society. The OECD’s Survey of Adult Skills, conducted as part of the Programme for the International Assessment of Adult Competencies (PIAACs), highlights this broader relevance by assessing literacy, numeracy, and problem-solving in technology-rich environments among adults aged 18–65. Data from the 2022–2023 survey in OECD countries revealed that roughly one-quarter of the population achieves only Level 1 (out of five) across all three competencies, reflecting challenges in basic cognitive and workplace skills (OECD, 2024). In contrast, higher proficiency levels are linked to enhanced employability, better health, and greater overall life satisfaction.

The psychological literature further emphasizes everyday cognitive competence as the ability to manage cognitively demanding tasks, often described as instrumental activities of daily living (IADLs). These tasks include using the telephone, preparing meals, housekeeping, managing medications, handling finances, and shopping—skills that are crucial for maintaining functional independence (Lawton & Brody, 1969). These activities occur in real-world environments, and cognitive processes account for approximately one-fifth of the variance in cognitively demanding functional task performance (Royall et al., 2007), indicating the significant role of non-cognitive factors in influencing such performance. While younger adults generally outperform older adults on tasks that require high cognitive demands (Borella et al., 2017; McAlister & Schmitter-Edgecombe, 2016), many older adults maintain functional independence despite measurable declines in standardized neuropsychological assessments. Factors such as lifestyle (Kalisch et al., 2011), self-efficacy (Helmes & Klinger, 2017), and social support systems (Whitfield & Wiggins, 2003) play a significant role in maintaining functional independence by encouraging an engagement in cognitively demanding tasks and offering cognitive stimulation, which are essential for building a cognitive reserve and enhancing cognitive plasticity. In the following paragraphs, we will elaborate upon how cognitive predictors, such as EF, and non-cognitive predictors, such as loneliness, contribute to everyday cognitive competence. We will also discuss the current approaches for assessing everyday cognitive competence.

Cognitive processes are essential for daily functioning, and optimal functioning would be challenging, if not impossible, without preserved cognitive abilities. For example, memory plays a crucial role in daily tasks such as remembering appointments, following routines, and recalling important information, all of which are essential for maintaining independence and effectively navigating daily life. While numerous cognitive processes have been examined in this context—such as fluid reasoning, WM, processing speed, and crystallized abilities (Borella et al., 2017), EFs stand out as particularly important for understanding everyday cognitive competence (Tomaszewski Farias et al., 2009). EFs refer to a broad set of mental processes that monitor and control goal-oriented thoughts and actions (Diamond, 2013). Among the numerous identified EF subprocesses, the three most commonly investigated are inhibition, updating, and task shifting (Miyake et al., 2000). Inhibition involves the deliberate suppression of automatic, dominant, or prepotent responses. Updating refers to the continuous monitoring and active manipulation of WM contents by rapidly adding or removing information. Task shifting, on the other hand, reflects the ability to flexibly switch between tasks, mental sets, or operations. Together, these three components represent distinct but interconnected dimensions of EF. The relationship between these executive processes and everyday cognitive competence is an important area of research for several reasons. First, basic EF processes serve as the foundation for higher-order cognitive abilities (Diamond, 2013). Second, deficits in these processes often emerge earlier than other age-related cognitive changes and can predict subsequent declines, making them critical markers of cognitive aging (Lacreuse et al., 2020). Finally, interventions aimed at enhancing cognitive abilities, by practicing core EF processes with the goal of generalizing their effects to untrained abilities and everyday life, can be successful. For example, cognitive training improved IADLs in patients with acquired brain injuries (Man et al., 2006) and led to a reduction in reported subjective cognitive lapses after a stroke (Westerberg et al., 2007).

Previous research has shown that EFs play a significant role in predicting everyday cognitive performance and are essential for managing complex daily activities (Jefferson et al., 2006). While all three core EF processes—updating, inhibition, and task shifting—have been linked to solving everyday complex problems, their contributions are not always consistent across studies. For instance, some studies highlight updating as a key predictor of everyday cognitive competence, whereas inhibition and shifting have been found to play less significant roles (e.g., McAlister & Schmitter-Edgecombe, 2016). In contrast, other studies have found that shifting has the strongest association with everyday cognitive competence (González-Andrade et al., 2022; Vaughan & Giovanello, 2010). Yet another perspective highlights that different EF components may be differentially associated with specific domains of daily functioning. For instance, inhibition and updating have been linked to declines in basic activities of daily living or fundamental self-care tasks (e.g., eating, dressing, bathing, and toileting), while shifting is more strongly associated with impairments in complex activities, such as IADLs (Verreckt et al., 2022). These findings suggest that while EF processes collectively underpin everyday cognitive competence, their specific contributions may vary depending on the type of daily activity and the demands placed on cognitive control mechanisms in administrated tasks.

In addition to cognitive factors, social networks can provide both tangible and intangible resources to perform well in cognitively demanding everyday tasks. Tangible support may include assistance with performing IADLs (such as assistance with meal preparation), while intangible support encompasses emotional encouragement, guidance that enhances motivation and confidence, and cognitive stimulation. Of particular importance is the examination of loneliness, which refers to the distress experienced when there is a mismatch between an individual’s preferred and actual social connections (Hawkley & Cacioppo, 2010). The adverse effects of loneliness are well documented and widely recognized in the literature. Loneliness is associated with an increased probability of stroke (Hakulinen et al., 2018), depression (Domènech-Abella et al., 2017), and dementia onset (Holwerda et al., 2014). The negative relations of loneliness and cognitive abilities and the performance of IADLs are also documented (Boss et al., 2015; Crewdson, 2016; Qi et al., 2023). The recent COVID-19 pandemic has highlighted the impact of social isolation and loneliness on everyday cognitive competence. During this period, older adults experienced a decline in transportation and shopping competence, areas directly affected by isolation measures. In contrast, no such effects were observed in domains like financial management or cooking, likely because these activities were less influenced by social restrictions (McArthur et al., 2022). These changes continue to shape older adults’ functioning in the post-pandemic period, as they now prefer outdoor activities over indoor gatherings, notably reducing their engagement with the arts, culture, and indoor recreational amenities, reflecting a broader shift in social and leisure preferences (Finlay et al., 2024).

Everyday cognitive competence is a complex construct, and measuring it is equally challenging. Ideally, an assessment would encompass the performance of ecologically valid tasks, as well as performance across various situations, environments, and over time. To achieve a comprehensive understanding, this construct can be evaluated through different methods, such as self-report questionnaires and performance-based tasks, each capturing distinct aspects of this multifaceted phenomenon. This distinction can be likened to the difference between a street and a topographic map—one guides you through the streets, while the other gives you a deeper understanding of the terrain. Self-report measures provide a practical and efficient method for capturing one’s experiences across a variety of real-world contexts. They provide valuable insights into individuals’ ratings of their performance in different areas of the IADLs (Lawton & Brody, 1969), as well as lapses in cognitive functioning throughout daily activities (Broadbent et al., 1982). These measures are particularly useful for assessing subjective perceptions of cognitive competence. However, their reliance on self-perception introduces the risk of reporting biases, which can manifest as the overestimation or underestimation of one’s abilities (Schmitter-Edgecombe et al., 2020). These biases may, in turn, stem from underlying cognitive deficits. For example, individuals with memory impairments might struggle to accurately recall instances of cognitive difficulties. Additionally, phenomena, such as negative self-evaluation, can increase the likelihood of identifying deficits, even when the objective performance might not support such assessments. In contrast, performance-based tasks offer a more objective means of evaluating everyday cognitive competence. These tasks require participants to solve standardized real-world problems, thereby simulating decision-making processes rather than relying on subjective reports. For instance, a performance-based task might involve filling out the form for selecting a phone service package. While these tasks are more time-consuming compared to self-report questionnaires, they provide a detailed evaluation of cognitive processes and skills, making them particularly sensitive to subtle intra- and interindividual differences (Tucker-Drob, 2011). Notably, these two assessment approaches demonstrate distinct patterns of association with other psychological and behavioral measures. Performance-based measures tend to be more strongly correlated with objective cognitive abilities, as they capture subtle differences in cognitive performance (McAlister & Schmitter-Edgecombe, 2016; Vaughan & Giovanello, 2010). In contrast, self-reports may show stronger associations with non-cognitive traits and states, as they tap more into participants’ self-evaluations (Wright & Osborne, 2005).

The aim of the study is to examine how core EF components—updating, inhibition, and task shifting—predict everyday cognitive competence, and to explore whether loneliness contributes uniquely to the prediction of everyday cognitive competence beyond the effects of EF processes. This study focuses on middle-aged adults (45 to 65 years old), a population still actively engaged in the workforce and generally expected to exhibit only light cognitive deficits. It is noteworthy that, although the adverse effects of loneliness are often studied in older adults in the context of aging, some evidence suggests that these negative effects can be observed as early as age 50 (Hawkley et al., 2010). Unlike the majority of prior research, which predominantly involves older adults (Willis, 1996), individuals with pathological conditions, such as mild cognitive impairment or dementia (Law et al., 2012), or special populations (e.g., individuals living with HIV; González-Andrade et al., 2022), this study examines a healthy population to provide insights into predictors of everyday cognitive competence in the earlier stages of cognitive aging. To account for the multifaced nature of everyday cognitive competence, this study adopts a comprehensive approach by including both performance-based assessments and self-reported measures.

## 2. Materials and Methods

### 2.1. Participants

The sample consisted of 176 middle-aged adults (mean age = 54.6, SD = 4.5, age range 43–65). We reached out to several companies and organizations, recruiting a convenient sample of individuals employed there. Participants were included based on the self-reported absence of the following: (1) psychiatric or neurological conditions, (2) use of medications for dementia, (3) significant visual or hearing impairments, and (4) other health issues that could hinder their ability to work. The sample comprised 67.4% women and varied in educational attainment, with 0.6% having completed elementary school, 36.5% high school, 10.1% a bachelor’s degree, and 53.4% holding a master’s degree or higher.

### 2.2. Materials

The Everyday Problems Test—brief version (EPT-BV; Juras et al., 2022) is a paper–pencil performance-based measure of everyday cognitive competence. The EPT-BV is an abbreviated and culturally adapted version of the original Everyday Problems Test (Willis & Marsiske, 1993) and consists of seven tasks, each related to one of the seven IADL domains (household care, medication use, meal preparation, shopping, telephone use, financial management, and transportation). In each task, an example of real-life material is used (such as an appliance manual or a book order form), and the participant’s task is to answer two open questions related to these materials. For example, participants are shown a book order form. Based on the information on the form, they are asked to answer two questions: how much would two specific books cost and which book would be the most suitable for a particular purpose? While the administration time is not limited, it typically takes approximately 10 min to complete the EPT-BV. The final score for each of the brief versions is the sum of the correct answers (maximum of 14).

The Cognitive Failures Questionnaire (CFQ; Wilhelm et al., 2010) is a 12-item self-report measure of the frequency of everyday cognitive lapses (minor cognitive errors, omissions, and lapses that disrupt the completion of daily tasks). Each item is rated on a scale ranging from 0 (never) to 4 (very often), where higher scores reflect a greater frequency of cognitive failures. The total score is calculated as the sum of ratings across all items with a high internal consistency (α = 0.88).

The N-back task (Jaeggi et al., 2010) is a computerized measure of WM updating. Participants must identify when the current stimulus—a consonant from a set of eight—matches one presented n position earlier in the sequence. Each stimulus is displayed for 500 milliseconds, followed by a 2500-millisecond interstimulus interval. The task includes a practice block for each difficulty level to familiarize participants with the task requirements. Following practice, participants complete 2 blocks of 1-back, 2-back, and 3-back difficulty levels, each containing 20+n stimuli. The task takes approximately 6 min to complete. Overall performance is calculated as the proportion of hits minus the proportion of false alarms averaged across all difficulty levels.

The Stroop task (Stroop, 1935) is a widely used measure of inhibition. In this task, participants are instructed to name the font color of a presented stimulus by pressing the corresponding key as quickly and accurately as possible. The stimulus can belong to one of three conditions: congruent (e.g., the word “BLUE” displayed in blue font), incongruent (e.g., the word “BLUE” displayed in red font), or neutral (e.g., a row of five asterisks displayed in one of four colors: red, green, yellow, or blue). In the current study, the task is divided into three blocks with 25%, 50%, and 75% congruent trials. Each block consisted of 40 trials (in total 120), and participants completed a practice block before performing the main task. It takes approximately 8 min to complete the task. The dependent measure is the difference in reaction time between congruent and incongruent trials (cost), based solely on correct responses.

The Local–Global task (LG; Navon, 1977) is a measure of task shifting. In this task, participants are presented with a large shape (global figure: circle, X, triangle, or square) composed of smaller shapes (local figure, e.g., large circle made of small triangles), displayed in either red or black. Importantly, the global and local figures are never the same shape. The task requires participants to count the number of lines in the global shape when the stimulus is black or the number of lines in the smaller shapes when the stimulus is red. Responses are made by pressing one of four keys on the keyboard. The task begins with two blocks in which all stimuli are either red or black. This is followed by a mix block where the stimulus color alternates. In half of the trials, the color remains the same as in the previous trial (non-switching), while in the other half, the color changes (switching). Participants complete practice block and a total of 48 experimental trials in the mixing block. It takes approximately 6 min to complete the task. The overall score is calculated as the difference in reaction times for correct responses between switching and non-switching trials (switching cost).

The UCLA Loneliness Scale (UCLA; Allen & Oshagan, 1995) is a 7-item self-report questionnaire that measures subjective feelings of loneliness. Participants rate each item on a 5-point Likert scale, ranging from 1 (does not apply to me at all) to 5 (applies to me completely). The responses were summed to produce a total score ranging from 7 to 35, with higher scores indicating a higher loneliness level, with a high internal consistency (α = 0.84).

### 2.3. Procedure

EF tasks were administered on participants’ personal computers, while the EPT, CFQ, and UCLA questionnaire were completed using a paper-and-pencil format at participants’ own pace. This study was approved by the Ethical Committee of the research institution and all participants gave their written informed consent in accordance with the Declaration of Helsinki.

### 2.4. Data Analysis

The data were analyzed using IBM SPSS Statistics version 25. For the Stroop and Local–Global tasks, the median reaction time for each condition and participant was calculated to compute cost measures. Outliers, defined as values exceeding ±3.29 standard deviations from the mean (z-scores > |3.29|), were excluded from analyses following the recommendations of Tabachnick et al. (2013). Two hierarchical regression analyses (HRAs) were conducted to explore the predictors of the EPT and CFQ. In the first step, sociodemographic variables (age, gender, and education) were entered as a predictor. In the second step, EF tasks performance was included (N-back, Stroop, LG task), and in the third step, loneliness, measured by the UCLA Loneliness Scale, was added.

## 3. Results

The descriptive statistics for the administered tests and tasks are presented in Table 1. The skewness and kurtosis values for all variables did not exceed two, indicating that the distributions are normal. The correlations between the observed variables are shown in Table 2. The performance on the EPT and self-reported scores on the CFQ were not correlated. Higher EPT scores were associated with higher levels of education and a higher performance on the N-back task. Women and participants who reported higher levels of loneliness also reported more cognitive lapses on the CFQ.

A hierarchical regression analysis was conducted to examine whether EF and loneliness can predict the EPT performance and CFQ ratings above sociodemographic variables (Table 3). For the EPT performance, age, gender, and education were included as predictors in the first step. Among these variables, only education made a significant independent contribution to predicting the EPT performance (β = 0.193, *p* < 0.05). However, the overall model did not reach statistical significance. In the second step, EF tasks were added, leading to a significant increase in the explained variance (ΔR^2^ = 0.043, ΔF = 2.7, *p* < 0.05). Of the three EF tasks, only the N-back performance significantly predicted the EPT performance (β = 0.176, *p* < 0.05). In the final step, loneliness (UCLA) was included but did not predict the EPT performance.

For the CFQ ratings, sociodemographic variables in the first step explained 10.5% of the variance, with gender being a significant predictor (β = 0.297, *p* < 0.01), indicating that women reported more cognitive lapses. In the second step, EF tasks were introduced, but they did not lead to a significant increase in the explained variance. In the third step, loneliness (UCLA) was added, which significantly improved the model (ΔR^2^ = 0.0076, ΔF = 16.1, *p* < 0.001). Loneliness emerged as a significant predictor of cognitive failures (β = 0.288, *p* < 0.01), suggesting that participants who reported higher feelings of loneliness also reported more cognitive lapses.

## 4. Discussion

The relationship between the performance on standardized cognitive tests and cognitive functioning in everyday life poses a challenge for experts across various fields, including psychology, gerontology, medicine, and occupational therapy. The understanding of this relationship is further complicated by the influence of non-cognitive factors, which can impact everyday cognitive competence beyond basic cognitive abilities. Numerous factors could be explored within this context; however, core EFs, due to their fundamental role in supporting higher-order cognitive processes, and loneliness—often referred to as an epidemic in contemporary society (Jeste et al., 2020)—could serve as valuable starting points for investigation. Therefore, this study explores how core EFs—updating, inhibition, and task shifting—predict everyday cognitive competence and whether loneliness adds unique explanatory value to this prediction. Our results indicate that the answers to these research questions depend on whether objective or subjective measures were used to assess everyday cognitive competence. Specifically, the nonsignificant relationship between the EPT and the CFQ, along with differing patterns of associations with sociodemographic variables, EF tasks, and loneliness, suggests that these two measures capture distinct aspects of everyday cognitive competence and offer complementary information.

A higher EPT score was associated with a better performance of updating tasks but showed no significant association with the performance of inhibition or task-shifting tasks. Updating, as a core EF, involves the active monitoring and manipulation of information in the WM, a process critical for solving complex problems, such as those encountered in the EPT. For example, successfully solving a multistep math problem (e.g., calculating the price of multiple articles in order) requires the constant updating of intermediate results or steps. Prior research also supports the idea that updating is the strongest predictor of fluid reasoning among the three core EF components (T. Chen & Li, 2007). Importantly, updating was the only EF component in our study assessed through accuracy, whereas inhibition and task shifting were measured via reaction times. This methodological distinction may partly explain the pattern of observed results. The EPT, being untimed, likely allowed participants to compensate for subtle deficits in inhibition and task shifting by taking additional time to process and respond. Such compensation may have diminished the observable contribution of these two EF components to the EPT performance. In contrast, for tests like the Timed Instrumental Activities of Daily Living (TIADL; Owsley et al., 2002), which simulates real-life actions across various domains, strict time constraints of 2–3 min per item impose higher demands on rapid decision-making and response execution. For such time-sensitive measures, inhibition and task shifting might play a more critical role in predicting outcomes, as there is a limited opportunity to compensate for deficits in these domains.

The performance of EF tasks did not predict self-reported cognitive lapses, and no significant correlation was found between the EPT and the CFQ. This lack of association aligns with previous research, which has similarly failed to identify significant relationships between CFQ scores and performance in cognitive tasks (Fastame, 2022; Könen & Karbach, 2020). It also reflects the generally limited correspondence between performance-based measures and the self-reported assessments of everyday cognitive competence (B. Chen et al., 2019; Schmitter-Edgecombe et al., 2011). The lack of this association may be influenced by several factors, which highlight key differences between self-reported and performance-based measures of cognitive function (Goodhew & Edwards, 2024). First, the CFQ measures a broader spectrum of cognitive functions, whereas EF tasks are designed to target specific, narrowly defined cognitive components, such as updating, inhibition, and shifting. This fundamental difference in scope may limit the comparability of CFQ scores with the more focused metrics of EF tasks. Second, self-report measures, like the CFQ, capture cognitive lapses over an extended period, typically the preceding six months, whereas objective EF tasks and the EPT performance are assessed at a single point in time. Moreover, the structured, standardized nature of performance-based tasks contrasts sharply with the unstructured and dynamic contexts of everyday life. Everyday situations often impose higher and more variable cognitive demands, increasing the likelihood of lapses that are not easily captured by objective tasks conducted in controlled environments. This contextual mismatch may have further contributed to the observed lack of association.

Loneliness, characterized as a distressing sense of perceived social isolation, has been associated with negative outcomes in physical, psychological, and cognitive health (Cacioppo et al., 2015). In our study, loneliness predicted (subjective) cognitive failures, but not the (objective) performance on the EPT. The mechanisms linking loneliness to cognitive performance include an decrease in social interactions with age, poorer sleep quality, and an increased cognitive load from hypervigilance (Cacioppo & Hawkley, 2009). Longitudinal studies indicate that sustained loneliness predicts a faster EF and memory decline in middle-aged and older adults (Tao et al., 2022). In contrast, transient loneliness —reported at one time but not the next—has been linked to a reduced risk of dementia, suggesting that the recovery from loneliness may enhance resilience against cognitive decline (Akhter-Khan et al., 2021). Interventions that promote group activities have demonstrated a reduction in loneliness and an improvement in cognitive function within three months (Pitkala et al., 2011), emphasizing the importance of addressing loneliness not only to support emotional well-being but also to preserve cognitive health and everyday functioning.

However, our findings also align with the notion that subjective cognitive failures may reflect emotional or psychological states (Könen & Karbach, 2020; Markett et al., 2020). Loneliness might predict mood, motivation, or stress levels, which could, in turn, mediate its relationship with subjective reports of cognitive failures. Individuals experiencing such states might be more prone to cognitive lapses and more likely to notice them. Indeed, both the UCLA loneliness scale and the CFQ are self-reported measures, and the association between the two could be confounded by methodological factors, such as response bias. Furthermore, this relationship may be explained by a third factor, such as personality traits. For example, studies have shown that neuroticism is linked to both CFQ (Könen & Karbach, 2020) and UCLA scores (Wang & Dong, 2018). Individuals with higher neuroticism may be more prone to perceive and report cognitive difficulties as well as levels of loneliness, which could explain the association between loneliness and subjective cognitive failures. This is further supported by the significant contribution of gender in predicting CFQ scores. Specifically, women reported experiencing cognitive failures more frequently than men, which aligns with findings from previous studies (e.g., Bridger et al., 2013; Volosin et al., 2023). Women tend to have a higher neuroticism which increases their awareness of cognitive errors (Carrigan & Barkus, 2016; Volosin et al., 2023). Furthermore, women often take on a larger share of cognitively demanding household responsibilities (Haupt & Gelbgiser, 2024). These tasks typically require multitasking, and with limited attentional capacity, this makes women more susceptible to various forms of cognitive failure (Iwasa et al., 2021).

Here, we should also consider that our sample consisted of healthy middle-aged adults, who, in their daily lives, face a variety of cognitively demanding tasks related to both professional responsibilities and family care. Given these challenges, it was expected that they would perform well on performance-based measures like the EPT. Indeed, our sample demonstrated generally strong performances. The tasks included in the EPT were specifically designed to be solvable by individuals with average cognitive abilities. Therefore, errors on the EPT likely reflect mistakes that could also occur in real-life situations, such as misinterpreting medication instructions or failing to follow directions for using technical devices. These errors should not be interpreted as major deficits, but rather as instances of mistakes that are common in everyday cognitive tasks requiring considerable attention and accuracy. However, we also observed meaningful individual differences in EPT performances. Education and updating emerged as significant predictors of the EPT performance, which is consistent with previous research (McAlister & Schmitter-Edgecombe, 2016) and PIAAC (OECD, 2024). While these predictors accounted for only a small proportion of the variance, this was expected given the relative homogeneity of our sample, especially considering that as individuals age, the variability in cognitive abilities and the rates of cognitive decline tend to become more pronounced. Furthermore, the processes of cognitive differentiation and dedifferentiation should also be taken into account. Cognitive differentiation, observed in younger adults, is characterized by weaker correlations across various cognitive tasks, indicating the presence of more specialized cognitive domains. In contrast, cognitive dedifferentiation in older adults is reflected in stronger correlations among different cognitive tasks, suggesting a reduced independence of cognitive domains as aging progresses (Baltes & Lindenberger, 1997). Consequently, the relationship between cognitive abilities and everyday cognitive competence is likely to be stronger in older populations, reflecting the cumulative effects of cognitive aging and the process of dedifferentiation.

The findings of this study emphasize that performance-based and self-report measures of everyday cognitive competence provide distinct yet complementary information. Performance-based measures, such as the EPT, are especially valuable when the research focus is on an individual’s ability to perform cognitively demanding tasks and should be administered in conjunction with self-reports. Even brief versions of the EPT can offer meaningful insights into everyday cognitive functioning, making them a practical tool and addressing concerns that such tests require substantial time for administration. In contrast, self-report measures, like the CFQ, provide valuable insights into individuals’ subjective assessments of their cognitive abilities. These self-reports are particularly useful for understanding how individuals perceive and navigate their cognitive strengths and limitations in real-world, dynamic environments. However, they may also reflect non-cognitive factors, such as emotional states or personal biases. Given these complementary roles, researchers and practitioners should carefully select the appropriate type of measure based on their specific research or clinical objectives. For studies focusing on cognitive capacities and problem-solving abilities, performance-based tools, like the EPT, are indispensable. On the other hand, when the aim is to understand subjective experiences or perceived cognitive difficulties, self-report measures offer essential context that cannot be captured by objective tests alone.

This study has several limitations that should be considered. First, participants were assessed at a single time point, which limits the ability to capture changes over time. It is possible that changes in EF or loneliness may predict everyday cognitive competence, rather than initial levels alone. Additionally, while we hypothesized that EF tasks would predict the subjective experiences of cognitive lapses, prior research suggests a reversed direction: self-reported cognitive lapses—particularly in older adults—may precede and predict future measurable declines in objective cognitive performance (Mitchell et al., 2014). Furthermore, the measures of everyday cognitive competence used in this study do not fully overlap. The EPT assesses the performance of tasks related to the instrumental activities of daily living (IADLs), while the CFQ focuses on various cognitive lapses in everyday life. These lapses, especially at mild levels, may not necessarily impair the ability to perform everyday tasks successfully. Although self-report measures of IADLs exist, they were not utilized in this study because they are designed to capture more pronounced deficits in the IADL performance. Given the characteristics of our sample, significant ceiling effects on the CFQ were unlikely, which contributed to its selection as the self-report measure. Lastly, a limitation of our study is the use of a convenience sample with a higher level of education compared to the general population. While this does not undermine the results, further research with more diverse samples is needed to enhance the generalization of the findings.

In conclusion, the extent to which EF and loneliness contribute to everyday cognitive competence in middle-aged adults depends on the specific aspect of this competence being evaluated. When focusing on the performance of objective everyday cognitive competence tasks, EF—particularly the ability to update information—emerges as a significant predictor. Conversely, loneliness does not appear to predict the performance of objective measures of everyday cognitive competence. When subjective experiences are the primary focus, the predictors differ and are not necessarily tied to the performance of standard cognitive tasks or objective measures. For instance, self-reported cognitive lapses are associated with feelings of loneliness, likely because both constructs reflect subjective experiences rather than objective abilities. Future research should carefully distinguish between these two aspects of everyday cognitive competence, as different predictors may uniquely influence each domain. This distinction is also important in the context of meta-analyses and systematic reviews, where recognizing these differences is important for the accurate integration and interpretation of findings.

## Figures and Tables

**Table 1:** Descriptive statistics for sociodemographic variables, everyday cognitive competence measures, EF tasks, and loneliness (N = 176).

	M	SD	Min	Max
EPT	11.7	1.7	6	14
CFQ	13.7	6.7	0	35
UCLA	13.1	4.7	5	31
N-back	0.66	0.16	0.16	0.95
Stroop	196	127	−81	634
Local–Global	160	264	−416	978

Note: EPT—Everyday Problems Test—brief version; CFQ—Cognitive Failures Questionnaire.

**Table 2:** Intercorrelations among sociodemographic variables, everyday cognitive competence measures, EF tasks, and loneliness (N = 176).

	1	2	3	4	5	6	7	8
1 Age	-							
2 Gender	−0.12	-						
3 Education	0.07	0.01						
4 EPT	0.06	0.05	0.20 **					
5 CFQ	−0.04	0.30 **	−0.13	−0.10				
6 N-back	0.03	−0.19 *	0.20 **	0.21 **	−0.07			
7 Stroop	0.12	0.10	0.06	−0.09	0.12	−0.16 *		
8 Local–Global	0.13	−0.07	−0.13	−0.01	0.11	−0.07	0.17 *	
9 UCLA	0.17 *	0.10	−0.18 *	−0.08	0.32 **	−0.07	0.01	0.04

Note: EPT—Everyday Problems Test—brief version; CFQ—Cognitive Failures Questionnaire. ** *p* < 0.01, * *p* < 0.05, and gender coding = male (1), female (2).

**Table 3:** Summary of hierarchical regression analyses for variables predicting EPT and CFQ score (N = 176).

	EPT			CFQ		
	1st Step (β)	2nd Step (β)	3rd Step (β)	1st Step (β)	2nd Step (β)	3rd Step (β)
Age	0.052	0.061	0.074	0.002	−0.024	−0.082
Gender	0.052	0.099	0.107	0.295 **	0.297 **	0.260 **
Education	0.193 **	0.170 *	0.157 *	−0.133	−0.129	−0.073
N-back		0.176 *	0.175 *		0.033	0.037
Stroop		−0.100	−0.102		0.087	0.096
Local–Global		0.044	0.044		0.110	0.110
UCLA			−0.065			0.288 **
R	0.209	0.295	0.301	0.323	0.356	0.450
R^2^	0.044	0.087	0.091	0.105	0.126	0.203
ΔR^2^	0.044	0.043	0.044	0.105	0.022	0.076
ΔF	2.6	2.7 *	0.7	6.9 ***	1.4	16.1 ***

Note: EPT—Everyday Problems Test—brief version; CFQ—Cognitive Failures Questionnaire. *** *p* < 0.001, ** *p* < 0.01, * *p* < 0.05, and gender coding = male (1), female (2).

## Data Availability

The raw data supporting the conclusions of this article will be made available by the authors on request.

## References

[B1-ejihpe-15-00058] Akhter-Khan S. C., Tao Q., Ang T. F. A., Itchapurapu I. S., Alosco M. L., Mez J., Piers R. J., Steffens D. C., Au R., Qiu W. Q. (2021). Associations of loneliness with risk of Alzheimer’s disease dementia in the Framingham heart study. Alzheimer’s & Dementia.

[B2-ejihpe-15-00058] Allen R. L., Oshagan H. (1995). The UCLA loneliness scale: Invariance of social structural characteristics. Personality and Individual Differences.

[B3-ejihpe-15-00058] Alloway T. P., Alloway R. G. (2013). Working memory across the lifespan: A cross-sectional approach. Journal of Cognitive Psychology.

[B4-ejihpe-15-00058] Baltes P. B., Lindenberger U. (1997). Emergence of a powerful connection between sensory and cognitive functions across the adult life span: A new window to the study of cognitive aging?. Psychology and Aging.

[B5-ejihpe-15-00058] Borella E., Cantarella A., Joly E., Ghisletta P., Carbone E., Coraluppi D., Piras F., De Beni R. (2017). Performance-based everyday functional competence measures across the adult lifespan: The role of cognitive abilities. International Psychogeriatrics.

[B6-ejihpe-15-00058] Boss L., Kang D.-H., Branson S. (2015). Loneliness and cognitive function in the older adult: A systematic review. International Psychogeriatrics.

[B7-ejihpe-15-00058] Bridger R. S., Johnsen S. Å., Brasher K. (2013). Psychometric properties of the cognitive Failures questionnaire. Ergonomics.

[B8-ejihpe-15-00058] Broadbent D. E., Cooper P. F., FitzGerald P., Parkes K. R. (1982). The Cognitive Failures Questionnaire (CFQ) and its correlates. British Journal of Clinical Psychology.

[B9-ejihpe-15-00058] Cacioppo J. T., Cacioppo S., Capitanio J. P., Cole S. W. (2015). The Neuroendocrinology of Social Isolation. Annual Review of Psychology.

[B10-ejihpe-15-00058] Cacioppo J. T., Hawkley L. C. (2009). Perceived Social Isolation and Cognition. Trends in Cognitive Sciences.

[B11-ejihpe-15-00058] Carrigan N., Barkus E. (2016). A systematic review of cognitive failures in daily life: Healthy populations. Neuroscience and Biobehavioral Reviews.

[B12-ejihpe-15-00058] Chen B., Huang Y., Wang D., Deng W. (2019). Comparison of performance-based observed assessment, self-report, and paper–pencil measures of everyday problem solving in Chinese older adults. Journal of Adult Development.

[B13-ejihpe-15-00058] Chen T., Li D. (2007). The roles of working memory updating and processing speed in mediating age-related differences in fluid intelligence. Aging, Neuropsychology, and Cognition.

[B14-ejihpe-15-00058] Crewdson J. A. (2016). The effect of loneliness in the elderly population: A review. Healthy Aging & Clinical Care in the Elderly.

[B15-ejihpe-15-00058] Diamond A. (2013). Executive Functions. Annual Review of Psychology.

[B16-ejihpe-15-00058] Domènech-Abella J., Lara E., Rubio-Valera M., Olaya B., Moneta M. V., Rico-Uribe L. A., Ayuso-Mateos J. L., Mundó J., Haro J. M. (2017). Loneliness and depression in the elderly: The role of social network. Social Psychiatry and Psychiatric Epidemiology.

[B17-ejihpe-15-00058] Fastame M. C. (2022). Are subjective cognitive complaints associated with executive functions and mental health of older adults?. Cognitive Processing.

[B18-ejihpe-15-00058] Ferguson H. J., Brunsdon V. E., Bradford E. E. (2021). The developmental trajectories of executive function from adolescence to old age. Scientific Reports.

[B19-ejihpe-15-00058] Finlay J., Meltzer G., O’Shea B., Kobayashi L. (2024). Altered place engagement since COVID-19: A multi-method study of community participation and health among older americans. Wellbeing, Space and Society.

[B20-ejihpe-15-00058] Glahn D. C., Kent J. W., Sprooten E., Diego V. P., Winkler A. M., Curran J. E., McKay D. R., Knowles E. E., Carless M. A., Göring H. H. H., Dyer T. D., Olvera R. L., Fox P. T., Almasy L., Charlesworth J., Kochunov P., Duggirala R., Blangero J. (2013). Genetic basis of neurocognitive decline and reduced white-matter integrity in normal human brain aging. Proceedings of the National Academy of Sciences.

[B21-ejihpe-15-00058] González-Andrade A., García-Torres A., Pérez-García M., Vergara-Moragues E. (2022). Assessment of executive functions as a measure of impairments in everyday functioning in persons with HIV. Psychology, Health & Medicine.

[B22-ejihpe-15-00058] Goodhew S. C., Edwards M. (2024). A meta-analysis on the relationship between subjective cognitive failures as measured by the cognitive failures questionnaire (CFQ) and objective performance on executive function tasks. Psychonomic Bulletin & Review.

[B23-ejihpe-15-00058] Hakulinen C., Pulkki-Råback L., Virtanen M., Jokela M., Kivimäki M., Elovainio M. (2018). Social isolation and loneliness as risk factors for myocardial infarction, stroke and mortality: UK Biobank cohort study of 479 054 men and women. Heart.

[B24-ejihpe-15-00058] Haupt A., Gelbgiser D. (2024). The gendered division of cognitive household labor, mental load, and family–work conflict in European countries. European Societies.

[B25-ejihpe-15-00058] Hawkley L. C., Cacioppo J. T. (2010). Loneliness matters: A theoretical and empirical review of consequences and mechanisms. Annals of Behavioral Medicine.

[B26-ejihpe-15-00058] Hawkley L. C., Thisted R. A., Masi C. M., Cacioppo J. T. (2010). Loneliness predicts increased blood pressure: Five-year cross-lagged analyses in middle-aged and older adults. Psychology and Aging.

[B27-ejihpe-15-00058] Helmes E., Klinger J. (2017). Prediction of everyday task performance in older adults by perceived health, self-efficacy and cognitive ability. Cogent Psychology.

[B28-ejihpe-15-00058] Holwerda T. J., Deeg D. J., Beekman A. T., Van Tilburg T. G., Stek M. L., Jonker C., Schoevers R. A. (2014). Feelings of loneliness, but not social isolation, predict dementia onset: Results from the Amsterdam Study of the Elderly (AMSTEL). Journal of Neurology, Neurosurgery & Psychiatry.

[B29-ejihpe-15-00058] Iwasa H., Yoshida Y., Ishii K., Yasumura S. (2021). Factors associated with cognitive failure among mothers involved in child care. Cogent Psychology.

[B30-ejihpe-15-00058] Jaeggi S. M., Buschkuehl M., Perrig W. J., Meier B. (2010). The concurrent validity of the *N*-back task as a working memory measure. Memory.

[B31-ejihpe-15-00058] Jefferson A. L., Paul R. H., Ozonoff A. L., Cohen R. A. (2006). Evaluating elements of executive functioning as predictors of instrumental activities of daily living (IADLs). Archives of Clinical Neuropsychology.

[B32-ejihpe-15-00058] Jeste D. V., Lee E. E., Cacioppo S. (2020). Battling the modern behavioral epidemic of loneliness: Suggestions for research and interventions. JAMA Psychiatry.

[B33-ejihpe-15-00058] Juras L., Martincević M., Vranić A., Rebernjak B., Hromatko I. (2022). The brief case for everyday problems: A proposal of two brief alternate forms of the Everyday Problems Test. European Journal of Ageing.

[B34-ejihpe-15-00058] Kalisch T., Richter J., Lenz M., Kattenstroth J.-C., Kolankowska I., Tegenthoff M., Dinse H. R. (2011). Questionnaire-based evaluation of everyday competence in older adults. Clinical Interventions in Aging.

[B35-ejihpe-15-00058] Könen T., Karbach J. (2020). Self-Reported Cognitive Failures in Everyday Life: A Closer Look at Their Relation to Personality and Cognitive Performance. Assessment.

[B36-ejihpe-15-00058] Lacreuse A., Raz N., Schmidtke D., Hopkins W. D., Herndon J. G. (2020). Age-related decline in executive function as a hallmark of cognitive ageing in primates: An overview of cognitive and neurobiological studies. Philosophical Transactions of the Royal Society B: Biological Sciences.

[B37-ejihpe-15-00058] Law L. L., Barnett F., Yau M. K., Gray M. A. (2012). Measures of everyday competence in older adults with cognitive impairment: A systematic review. Age and Ageing.

[B38-ejihpe-15-00058] Lawton M. P., Brody E. M. (1969). Assessment of older people: Self-maintaining and instrumental activities of daily living. The Gerontologist.

[B39-ejihpe-15-00058] Man D. W., Soong W. Y. L., Tam S. F., Hui-Chan C. W. (2006). A randomized clinical trial study on the effectiveness of a tele-analogy-based problem-solving programme for people with acquired brain injury (ABI). NeuroRehabilitation.

[B40-ejihpe-15-00058] Markett S., Reuter M., Sindermann C., Montag C. (2020). Cognitive failure susceptibility and personality: Self-directedness predicts everyday cognitive failure. Personality and Individual Differences.

[B41-ejihpe-15-00058] McAlister C., Schmitter-Edgecombe M. (2016). Executive function subcomponents and their relations to everyday functioning in healthy older adults. Journal of Clinical and Experimental Neuropsychology.

[B42-ejihpe-15-00058] McArthur C., Faller-Saunders A., Turcotte L. A., Sinn C.-L. J., Berg K., Morris J. N., Hirdes J. P. (2022). Examining the effect of the first wave of the COVID-19 pandemic on home care recipients’ instrumental activities of daily living capacity. Journal of the American Medical Directors Association.

[B43-ejihpe-15-00058] Mitchell A. J., Beaumont H., Ferguson D., Yadegarfar M., Stubbs B. (2014). Risk of dementia and mild cognitive impairment in older people with subjective memory complaints: Meta-analysis. Acta Psychiatrica Scandinavica.

[B44-ejihpe-15-00058] Miyake A., Friedman N. P., Emerson M. J., Witzki A. H., Howerter A., Wager T. D. (2000). The unity and diversity of executive functions and their contributions to complex “frontal lobe” tasks: A latent variable analysis. Cognitive Psychology.

[B45-ejihpe-15-00058] Navon D. (1977). Forest before trees: The precedence of global features in visual perception. Cognitive Psychology.

[B46-ejihpe-15-00058] OECD (2024). Do adults have the skills they need to thrive in a changing world? Survey of adult skills 2023.

[B47-ejihpe-15-00058] Owsley C., Sloane M., McGwin G., Ball K. (2002). Timed instrumental activities of daily living tasks: Relationship to cognitive function and everyday performance assessments in older adults. Gerontology.

[B48-ejihpe-15-00058] Pitkala K. H., Routasalo P., Kautiainen H., Sintonen H., Tilvis R. S. (2011). Effects of socially stimulating group intervention on lonely, older people’s cognition: A randomized, controlled trial. The American Journal of Geriatric Psychiatry.

[B49-ejihpe-15-00058] Qi X., Belsky D. W., Yang Y. C., Wu B. (2023). Association between types of loneliness and risks of functional disability in older men and women: A prospective analysis. The American Journal of Geriatric Psychiatry.

[B50-ejihpe-15-00058] Royall D. R., Lauterbach E. C., Kaufer D., Malloy P., Coburn K. L., Black K. J. (2007). The cognitive correlates of functional status: A review from the committee on research of the American neuropsychiatric association. The Journal of Neuropsychiatry and Clinical Neurosciences.

[B51-ejihpe-15-00058] Salthouse T. A. (2004). What and when of cognitive aging. Current Directions in Psychological Science.

[B52-ejihpe-15-00058] Schmitter-Edgecombe M., Parsey C., Cook D. J. (2011). Cognitive correlates of functional performance in older adults: Comparison of self-report, direct observation, and performance-based measures. Journal of the International Neuropsychological Society.

[B53-ejihpe-15-00058] Schmitter-Edgecombe M., Sumida C., Cook D. J. (2020). Bridging the gap between performance-based assessment and self-reported everyday functioning: An ecological momentary assessment approach. The Clinical Neuropsychologist.

[B54-ejihpe-15-00058] Stroop J. R. (1935). Studies of interference in serial verbal reactions. Journal of Experimental Psychology.

[B55-ejihpe-15-00058] Tabachnick B. G., Fidell L. S., Ullman J. B. (2013). Using multivariate statistics.

[B56-ejihpe-15-00058] Tao Q., Akhter-Khan S. C., Ang T. F. A., DeCarli C., Alosco M. L., Mez J., Killiany R., Devine S., Rokach A., Itchapurapu I. S., Zhang X., Lunetta K. L., Steffens D. C., Farrer L. A., Greve D. N., Au R., Qiu W. Q. (2022). Different loneliness types, cognitive function, and brain structure in midlife: Findings from the Framingham Heart Study. eClinicalMedicine.

[B57-ejihpe-15-00058] Tomaszewski Farias S., Cahn-Weiner D. A., Harvey D. J., Reed B. R., Mungas D., Kramer J. H., Chui H. (2009). Longitudinal changes in memory and executive functioning are associated with longitudinal change in instrumental activities of daily living in older adults. The Clinical Neuropsychologist.

[B58-ejihpe-15-00058] Tucker-Drob E. M. (2011). Neurocognitive functions and everyday functions change together in old age. Neuropsychology.

[B59-ejihpe-15-00058] Vaughan L., Giovanello K. (2010). Executive function in daily life: Age-related influences of executive processes on instrumental activities of daily living. Psychology and Aging.

[B60-ejihpe-15-00058] Verreckt E., Grimm E., Agrigoroaei S., de Saint Hubert M., Philippot P., Cremer G., Schoevaerdts D. (2022). Investigating the relationship between specific executive functions and functional decline among community-dwelling older adults: Results from a prospective pilot study. BMC Geriatrics.

[B61-ejihpe-15-00058] Volosin M., Hallgató E., Csábi E. (2023). Validation of the Hungarian version of the Cognitive Failures Questionnaire (CFQ). Heliyon.

[B62-ejihpe-15-00058] Wang B., Dong X. (2018). The Association Between Personality and Loneliness: Findings From a Community-Dwelling Chinese Aging Population. Gerontology and Geriatric Medicine.

[B63-ejihpe-15-00058] Westerberg H., Jacobaeus H., Hirvikoski T., Clevberger P., Östensson M. L., Bartfai A., Klingberg T. (2007). Computerized working memory training after stroke—A pilot study. Brain Injury.

[B64-ejihpe-15-00058] Whitfield K. E., Wiggins S. (2003). The influence of social support and health on everyday problem solving in adult African Americans. Experimental Aging Research.

[B65-ejihpe-15-00058] Wilhelm O., Witthöft M., Schipolowski S. (2010). Self-reported cognitive failures. Journal of Individual Differences.

[B66-ejihpe-15-00058] Willis S. L. (1996). Everyday cognitive competence in elderly persons: Conceptual issues and empirical findings. The Gerontologist.

[B67-ejihpe-15-00058] Willis S. L., Marsiske M. (1993). Manual for the everyday problems test.

[B68-ejihpe-15-00058] Wright D. B., Osborne J. E. (2005). Dissociation, cognitive failures, and working memory. The American Journal of Psychology.

